# Psychosocial Burden of Women With Cervical Intraepithelial Neoplasia in Japan: Results of A Web‐Based Survey

**DOI:** 10.1111/jog.70258

**Published:** 2026-03-30

**Authors:** Kayo Sato, Nobuyuki Oshima, Mamiko Onuki, Koji Matsumoto, Kotoba Okuyama

**Affiliations:** ^1^ MSD K.K Tokyo Japan; ^2^ Department of Obstetrics and Gynecology Showa University School of Medicine Tokyo Japan

**Keywords:** cervical lesions, human papillomavirus, Japan, online survey, psychosocial burden

## Abstract

**Aim:**

Human papillomavirus (HPV) infection carries a high risk of developing cervical intraepithelial neoplasia (CIN) and cancer in women. For women with CIN, follow‐up examinations and treatment are necessary and pose a psychosocial burden. This study investigated the psychosocial burden among women with CIN in Japan.

**Methods:**

This observational, cross‐sectional, online survey enrolled women aged 20–49 years in Japan who used a mobile health app (Kencom) linked to an employment‐based health insurance association database. Participants completed a screening questionnaire on cervical screening history and CIN status. Eligible women then completed the HPV Impact Profile (HIP) to assess their psychosocial burden. CIN status was confirmed using diagnostic codes recorded in the Kencom database.

**Results:**

A total of 404 women were eligible for the study, comprising 121 with confirmed CIN and 283 without CIN. The baseline characteristics were similar, although women with CIN were slightly younger (39.8 vs. 41.5 years) and less likely to be married (55.4% vs. 70.0%). Women with CIN had significantly higher total HIP scores, indicating greater burden than those without CIN (42.2 vs. 24.5; *p* < 0.001). The burden was evident in women with CIN grade 1 (*p* < 0.001) and more pronounced in younger women (trend for age groups; *p* < 0.001).

**Conclusions:**

This study is the first to report a significant psychosocial burden of CIN in Japan, irrespective of CIN severity, with a greater impact in younger women. This psychosocial burden can be alleviated by HPV vaccination with evidence of preventing CIN.

## 1 Introduction

Human papillomavirus (HPV) is a double‐stranded DNA virus that is associated with cervical cancers or dysplasia [1]. HPV infection is one of the most common sexually transmitted infections, and nearly all sexually active people will experience HPV infection during their lifetime [2] Most HPV infections are asymptomatic and resolve spontaneously within 2 years [2]. However, persistent infections may result in dysplasia, which is diagnosed as cervical intraepithelial neoplasia (CIN) and graded as CIN1–CIN3 [3]. CIN1 is a low‐grade squamous intraepithelial lesion, whereas CIN2 and CIN3 or high‐grade squamous intraepithelial lesions (HSIL) are considered pre‐cancerous. Most CIN1 and CIN2 lesions regress within 1 year, but some progress to CIN3 [3, 4] CIN2 and CIN3 lesions may lead to invasive cervical cancer [3].

The management and treatment of CIN are dependent on its initial grade. According to Japanese clinical guidelines, women with CIN1 or CIN2 should undergo regular follow‐up with cytology (and colposcopy if needed) every 6 months to monitor the lesions for CIN1, or cytology and colposcopy every 3–6 months for CIN2 [5, 6] Treatments, such as cervical conization procedure or laser vaporization, are recommended for women with CIN3 and some women with CIN2 [5, 6] Women treated for CIN2 or CIN3 should be followed up until remission is confirmed by cytology because the risk of invasive cancer is not completely eliminated. In fact, in the United States, it was suggested that surveillance should continue for ≥ 25 years (at 3 year intervals) following surgical treatment [7].

CIN places a considerable burden on affected women, not only in terms of the disease itself but also the medical procedures, which include screening, follow‐up assessments, and treatment, that may be continued for many years. Several studies have clearly demonstrated this increased psychosocial burden among women with CIN and people with other HPV‐related diseases [8, 9, 10, 11, 12, 13]. To our knowledge, however, no studies have investigated the psychosocial impact of CIN in women in Japan. We hypothesized that the psychosocial burden of women with CIN would exceed that of women without CIN. To test this hypothesis, we performed a cross‐sectional survey of women with or without CIN in Japan, and examined their psychosocial burden using the HPV Impact Profile (HIP), a 29‐item questionnaire across seven domains of HPV‐related health conditions [11].

## 2 Methods

### 2.1 Ethics

This observational, cross‐sectional study adhered to the Japanese Ethical Guidelines for Medical and Biological Research Involving Human Subjects and the Act on the Protection of Personal Information. This study was approved by the Toukeikai Kitamachi Clinic Ethics Review Board (approval number: MSD08190; date: July 21, 2021) and registered on the University Hospital Medical Information Network Clinical Trial Registry (UMIN000044717).

### 2.2 Participants and Survey Design

Women aged 20–49 years in Japan who used an online health service (Kencom, DeSC Healthcare Inc.) were invited to participate in this survey, which was advertised through online banner displays, mail magazine, and push notifications. Kencom is an online service available to members of employment‐based health insurance associations, which allow DeSC Healthcare to link questionnaire data to health insurance claims data and health check‐up data, and the data are subsequently deidentified. This service has been used in prior studies [14]. DeSC Healthcare covers approximately 550 000 insured individuals, and the Kencom application was used by approximately 180 000 registered users in 2023.

Between September 2021 and February 2022, invitations were sent in three waves to registered users, directing them to the survey in the Kencom application. The introductory page contained information about the aims and design of the survey. The participants were required to review the explanation and check a box on the informed consent page to confirm their consent to participate.

After confirming consent, the participants completed a screening questionnaire, which recorded the following: gender; age; self‐reported outcome of cervical cancer screening within the past 6 months; self‐reported hospital or clinic visits for the treatment of cervical abnormalities within the past 6 months; self‐reported HPV vaccination status; pregnancy; marital status; childbirth experience; and awareness of the HPV vaccine (HPV vaccine or cervical cancer vaccine).

Using the self‐reported information collected by the screening questionnaire, the participants were divided into two groups as follows: (1) women who had visited a hospital or clinic for the treatment of any cervical abnormalities up to 6 months before the survey (i.e., women with cervical abnormalities) and (2) women who had not visited a hospital or clinic for the treatment of HPV‐related cervical abnormalities up to 6 months before the survey (including “could not remember” or “unknown”) (i.e., women with normal cytology).

We also retrieved the participant's medical history over the 6 months prior to the survey from the linked administrative data (12 month look‐back to identify the number of visits in the CIN group) to confirm the presence or absence of CIN. These data were reviewed to identify the presence or absence of diagnoses or treatments based on the International Classification of Diseases (10th revision; ICD‐10) codes listed in Table S1. The codes listed in that table were also applied to exclude women with CIN or other diseases potentially related to HPV from the group of women without CIN.

Women were excluded from the analyses for the following reasons: (a) history of any type of HPV vaccination, (b) unknown HPV vaccination status, (c) abnormal pregnancy or unknown results for cervical cancer screening, or (d) providing the same response to all questions in the HIP.

All participants who completed the questionnaire received points that could be redeemed as an Amazon gift card worth 1000 yen (approximately US$6.75) as an incentive.

### 2.3 Assessment of Psychosocial Burden and Medical History

Women who satisfied the eligibility criteria, based on the information collected via the screening questionnaire, were asked to complete the Japanese versions of the HIP questionnaire and the Kessler 6 Psychological Distress Scale (K6).

The HIP questionnaire [11], was used to measure the psychosocial impact of HPV‐related health conditions in women. This questionnaire contains 29 items, all of which are rated on a discrete visual analog scale (VAS) ranging from 0 (no impact) to 10 points (worst impact) across seven domains: Worries and concerns, Emotional impact, Sexual impact, Self‐image, Partner issues and transmission, Interactions with doctors, and Control‐life impact. The dimension scores and the total burden score are all summarized as a range from 0 to 100, where higher scores represent a greater psychosocial impact of HPV. Scores were categorized as “no or little impact” (< 40), “moderate impact” (40–70), or “severe psychological impact” (> 70). The Japanese version of the HIP questionnaire was prepared and field‐tested by the Mapi Institute (https://www.mapi‐institute.com/).

The K6 scale measures the levels of distress and severity associated with psychological symptoms [15], for the following six items: (1) so sad nothing can cheer up; (2) nervous; (3) restless or fidgety; (4) hopeless; (5) everything was an effort; and (6) worthless. Each item is scored on a five‐point scale: 4 = all of the time; 3 = most of the time; 2 = some of the time; 1 = a little of the time; and 0 = none of the time. The total K6 score ranges from 0 to 24 and is categorized as low risk of distress (0–7), moderate risk of distress (7–12), or high risk of distress (13–24) [16, 17]. In this study, we used the Japanese version of the K6 scale [18] and scores divided into low risk of distress (0–7) or moderate/high risk of distress (8–26).

To our knowledge, no prior studies have reported the psychosocial impact of disease using the HIP questionnaire in Japan. Therefore, we also used a general scale of distress, the K6 scale, to confirm the results obtained with the HIP questionnaire, which is expected to be more sensitive for assessing the psychosocial impact of HPV‐related genital diseases than general scales [11].

### 2.4 Statistical Analyses

A formal sample size was not specified due to the descriptive design of the study. We estimated that 450 women with cervical abnormalities and 300 women with normal cytology would complete the survey, based on the following assumptions. Of approximately 150 000 individual members of the Kencom application, approximately 24 000 were aged 20–49 years (January 2021). Considering the previously published screening rates of cervical cancer among people aged 20–49 years in 2019 in Japan that ranged from 12.9% to 27.6% [19], we estimated that 700 women would have cervical abnormalities and 3000–6000 would have normal cytology. We also considered a response rate of 20% for the general population, a response rate of 50% for women with CIN, and a dropout rate of 30% due to an inability to link self‐reported CIN and diagnosis in the administrative database. Previous studies using HIP enrolled 200–310 women with cervical abnormalities and 50–241 women with normal cytology [9, 10, 12].

Data were analyzed for all eligible participants. The total scores and scores for each domain/item of the HIP [11] and K6 [15] questionnaires were calculated using the respective established methods. The outcomes were analyzed using descriptive statistics to determine the mean and standard deviation (SD) for continuous variables, and the number and percentage of participants for categorical variables. The HIP scores, total and individual domain scores, and proportions of women with moderate/severe burden (defined as HIP scores of ≥ 40) were compared between women with and without CIN using *t*‐tests and chi‐square tests. The total HIP and domain scores were also compared among women divided by disease severity (none; mild [CIN1]); moderate/severe [CIN2/3]; or unknown and by age (20–29, 30–39, and 40–49 years old [y.o.]). In women with CIN, trend tests were performed for the total and domain HIP scores across age groups. Owing to the skewed distribution, K6 scores were analyzed as a categorical variable, defined as the proportions of women with low risk of distress (i.e., scores 0–7) or moderate/high risk of distress (scores of 8–24), and compared between women with or without CIN using the chi‐square test. K6 total scores were also compared using *t*‐tests. SAS version 9.4 (SAS Institute Inc., Cary, NC, USA) was used for data analyses.

## 3 Results

### 3.1 Participants

**FIGURE 1:**
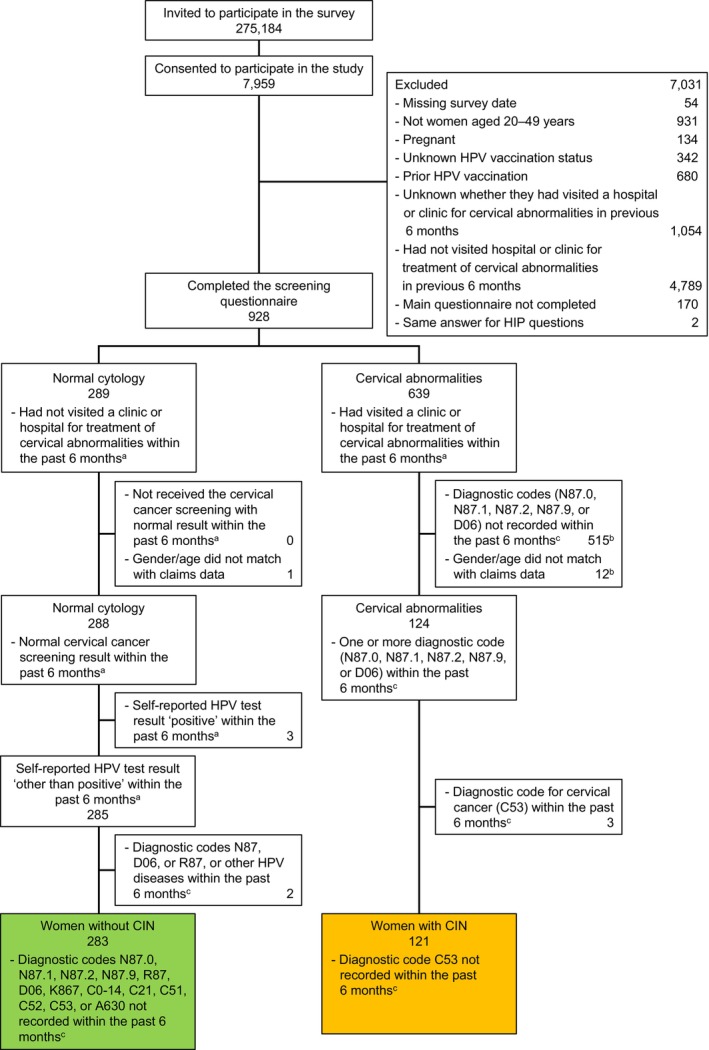
Patient disposition (a). Based on self‐reported responses from the screening questionnaire (b). Multiple reasons apply (c). Based on administrative claims data. CIN, cervical intraepithelial neoplasia; HIP, Human Papillomavirus Impact Profile; HPV, human papillomavirus.

A total of 275 184 invitations to participate in the survey were sent; 7959 people consented to participate, and 6974 women aged 20–49 years responded to the screening questionnaire (Figure 1). Of 928 women who successfully completed the screening questionnaire, 289 reported that they had normal cytology and 639 reported that they had cervical abnormalities. After reviewing their eligibility by reconciling the responses with the administrative data, 121 women with CIN and 283 women without CIN were eligible for this study.

The characteristics of women with CIN and women without CIN were generally similar in terms of prior childbirth experience (55.4% vs. 54.8%, respectively) and history of mental health consultations (3.3% vs. 2.5%), although women with CIN were slightly younger (mean ± SD; 39.8 ± 6.3 vs. 41.5 ± 5.8 years, *p* < 0.05) and less likely to be married (55.4% vs. 70.0%, *p* < 0.01). Regarding HPV vaccine awareness among women with CIN, 66.1% were aware of the name only and 27.3% knew about the vaccine well. Among women without CIN, the corresponding percentages were 70.3% and 24.7% (Table 1).

**TABLE 1:** Characteristics of women without CIN and women with CIN.

		Women without CIN	Women with CIN	*P* ^a^
*N*		283	121	
Age, years	Mean ± SD	41.5 ± 5.8	39.8 ± 6.3	0.011
Age category, years	20–29 y.o.	10 (3.5)	9 (7.4)	0.109
	30–39 y.o.	82 (29.0)	41 (33.9)	
	40–49 y.o.	191 (67.5)	71 (58.7)	
Marital status/partner	Yes	198 (70.0)	67 (55.4)	0.005
	No	84 (29.7)	53 (43.8)	
	Missing	1 (0.4)	1 (0.8)	
Prior childbirth	Yes	155 (54.8)	67 (55.4)	0.940
	No	127 (44.9)	54 (44.6)	
	Missing	1 (0.4)	0	
History of mental health consultation	Yes	7 (2.5)	4 (3.3)	0.638
	No	276 (97.5)	117 (96.7)	
HPV vaccine awareness (HPV vaccine or cervical cancer vaccine)	Yes (know well)	70 (24.7)	33 (27.3)	0.332
	Yes (only name)	199 (70.3)	80 (66.1)	
	No	10 (3.5)	8 (6.6)	
	Missing	4 (1.4)	0	
Severity of cervical disease	CIN1	—	17 (14.1)	—
	CIN2/3	—	42 (34.7)	
	Unknown	—	62 (51.2)	
Number of visits with claims data for HPV‐related disease in the previous 12 months	≤ 2 visits	—	44 (36.4)	—
	3 or 4 visits	—	43 (35.5)	
	≥ 5 visits	—	34 (28.1)	
Interval between the most recent visit and the survey	≤ 1 month	—	59 (48.8)	—
	2–3 months	—	37 (30.6)	
	≥ 4 months	—	25 (20.7)	

*Note:* Values are reported as *n* (%) of participants, except for age.

Abbreviations: CIN, cervical intraepithelial neoplasia; HPV, human papillomavirus; SD, standard deviation; y.o., years old.

^a^ Chi‐square test except for age (*t*‐test).

Among 121 women with CIN, the severity was classified as CIN1 in 17 (14.1%), CIN2/3 in 42 (34.7%), and unknown in 62 (51.2%). Overall, 44 (36.4%) had attended ≤ 2 visits, 43 (35.5%) had attended 3 or 4 visits, and 34 (28.1%) had attended ≥ 5 visits within 12 months before completing the survey. The interval between the most recent visit and the survey was ≤ 1 month for 59 (48.8%) women, 2–3 months for 37 (30.6%), and ≥ 4 months for 25 (20.7%).

### 3.2 Psychological Burden of CIN

**FIGURE 2:**
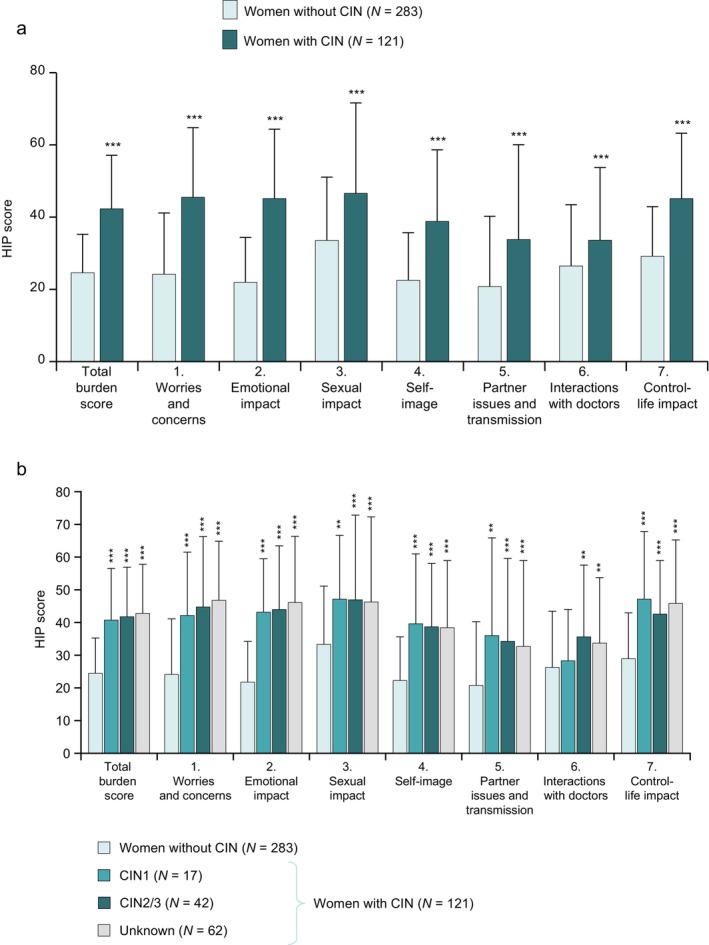
HIP scores in women with or without CIN. The total HIP scores and individual domain scores were compared between women with or without CIN (a), and among women without CIN and women with CIN divided by the severity of CIN (b). **p < 0.01 and ***p < 0.001 vs. women without CIN (chi‐square test). CIN, cervical intraepithelial neoplasia; HIP, Human Papillomavirus Impact Profile.

Figure 2a shows the total HIP scores and individual domain scores reported by 121 women with CIN and 283 women without CIN. Women with CIN reported worse HIP, with a higher total HIP score, than women without CIN (42.2 ± 14.9 vs. 24.5 ± 10.7, respectively; *p* < 0.001). The differences between women with and without CIN were particularly greater for the domains Emotional impact, Worries and concerns, Self‐image, and Control‐life impact (all *p* < 0.001). Furthermore, higher percentages of women with CIN had total HIP scores indicative of moderate or severe burden (moderate: 48.8%; severe: 3.3%; total moderate + severe: 52.1%) than women without CIN (moderate: 9.9%; severe: 0%; total moderate + severe: 9.9%), with similar findings for the individual domains (all *p* < 0.05) (Table 2).

**TABLE 2:** Proportions of women with moderate or severe psychosocial burden.

	Women without CIN	Women with CIN	*P* ^a^
*N*	283	121	
Moderate/severe burden: HIP score ≥ 40			
Total burden score	28 (9.9)	63 (52.1)	< 0.001
Worries and concerns	52 (18.4)	73 (60.3)	< 0.001
Emotional impact	32 (11.3)	74 (61.2)	< 0.001
Sexual impact	111 (39.2)	78 (64.5)	< 0.001
Self‐Image	27 (9.5)	52 (43.0)	< 0.001
Partner issues and transmission	37 (13.1)	45 (37.2)	< 0.001
Interactions with doctors	53 (18.7)	41 (33.9)	0.001
Control/life impact	60 (21.2)	70 (57.9)	< 0.001
Moderate/high risk of distress: K6 score ≥ 8	72 (25.4)	45 (37.2)	0.017

*Note:* Values are reported as *n* (%) of participants.

Abbreviations: CIN, cervical intraepithelial neoplasia; HIP, Human papillomavirus Impact Profile; K6, Kessler 6 Psychological Distress Scale.

^a^ Chi‐square test.

### 3.3 Psychological Burden According to the Severity of CIN

We also performed exploratory analyses to compare the total HIP scores and HIP domain scores according to the presence and severity of CIN: (a) women without CIN, (b) women with CIN1, (c) women with CIN2/3, and (d) women with an unknown severity of CIN (CIN unknown). This analysis showed that even CIN1 had a significant impact on the total HIP score compared with women without CIN (40.7 ± 15.8 vs. 24.5 ± 10.7; *p* < 0.001) (Figure 2b). Similarly, the total HIP score was significantly greater among women with CIN2/3 (total score: 41.8 ± 15.1; *p* < 0.001) and women with an unknown severity of CIN (42.9 ± 14.8; *p* < 0.001). The mean scores for most of the HIP domains were also significantly higher in these three subgroups than in women without CIN.

### 3.4 Psychological Burden According to the Age of the Women

**FIGURE 3:**
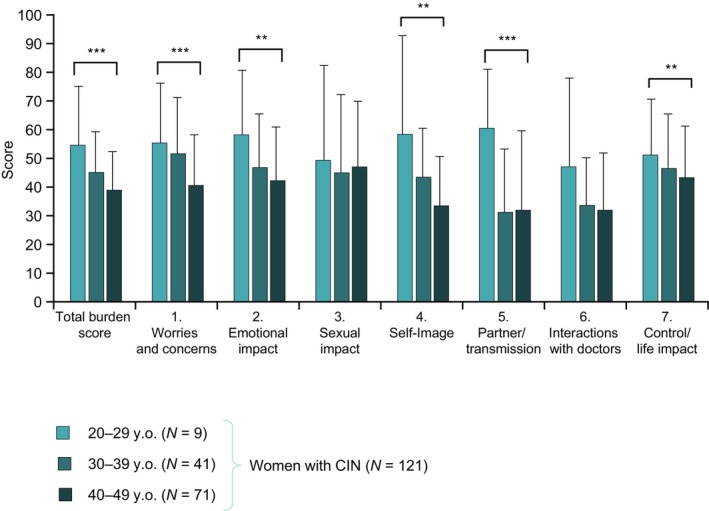
Total HIP scores and domain scores among women with CIN divided by age. Values are mean ± standard deviation. **p < 0.01 and ***p < 0.001 (trend test). CIN, cervical intraepithelial neoplasia; HIP, Human Papillomavirus Impact Profile; y.o., years old.

We performed age‐stratified analyses of the total HIP scores and the domain scores for the subgroup of women with CIN. As shown in Figure 3, the trend test across the age groups showed a significant trend toward greater scores in the younger age groups for the total HIP score and most of the domain scores, except for “Sexual impact” and “Interactions with doctors.” The pairwise comparisons indicated that the total HIP scores (mean ± SD) were significantly greater for the 20–29 y.o (54.6 ± 20.6) and 30–39 y.o (45.1 ± 14.2) groups than the 40–49 y.o. Group (38.9 ± 13.5; *p* < 0.01 vs. 20–29 y.o.; *p* < 0.05 vs. 30–39 y.o). We also found a particularly higher psychosocial burden related to “Self‐image” and “Partner issues and transmission” for the 20–29 y.o. Group compared with the other age groups.

### 3.5 Risk of Distress

The total K6 score was used as an index of the level of distress. A greater proportion of women with CIN reported moderate/high risk of distress compared with women without CIN (37.2% vs. 25.4%, respectively; *p* = 0.017) (Table 2). The mean (95% CI) K6 total score was 5.05 (4.46–5.65) among women without CIN, 4.88 (2.77–6.99) among women with CIN1 (*p* = 0.89 vs. women without CIN), and 7.24 (5.26–9.22) among women with CIN2/3 (*p* = 0.013 vs. women without CIN).

## 4 Discussion

This study was performed to investigate the perceived psychosocial burden of CIN among women in Japan. To our knowledge, this is the first such study in Japan. As we hypothesized, the general psychosocial burden, ascertained using the HIP, was significantly worse among women with CIN than among women without CIN. A notable finding is that this psychosocial burden was apparent even among women with mild cervical dysplasia (CIN1) and was similar to that of women with more severe cervical dysplasia (CIN2/3). Furthermore, the psychosocial burden was generally more pronounced in the 20–29 and 30–39 y.o. Groups than in the 40–49 y.o. Group.

The higher HIP scores in women in the 20–29 y.o. Group, with or without CIN, could be explained as follows. Women in the 20–29 y.o. Group reported higher scores for emotional impact of HIP, Self‐image, and partner‐related issues/infection, reflecting greater concern about future pregnancy and childbirth, compared with older women. Additionally, younger individuals have less prior experience of childbirth and screening, which may have resulted in a higher sense of burden from gynecological examinations. Nevertheless, some caution is needed when interpreting the results considering the number of women in the 20–29 y.o. Group (9 in the CIN group and 10 in the non‐CIN group). It is also important to consider that although HPV infection and early cervical lesions are more prevalent among women in their 20s [20], many of these infections are transient and resolve spontaneously without progressing to clinically significant disease [4]. Despite this favorable natural history, the processes of screening, diagnostic evaluation, and follow‐up may impose a substantial psychological burden, particularly among younger women.

Although the clinical burden of HPV in Japan was previously explored in terms of the incidence of CIN, no studies have examined its psychosocial burden. Therefore, we must discuss our findings in the context of studies in other countries, where differences in clinical practice or general attitudes toward vaccination might exist. Although Japan is a country with uniquely low vaccination rates compared with other countries, most previous studies and the present study only included HPV‐unvaccinated women and the HIP scores were similar across the studies. For example, our findings are broadly consistent with those of studies performed in other countries of women with CIN1 or CIN2/3 in South Korea (women with HPV disease: 53.37) [10] Taiwan (CIN1/2/3: 47.5) [13] the US (CIN1: 45.6; CIN2/3: 42.7) [11]. Australia (CIN1: 41.7; CIN2/3: 46.6) [12], and the United Kingdom (UK) (CIN1: 41.6; CIN2/3: 44.3) [9]. Similar to these earlier studies, we found that the psychosocial burden was already high among women with mild cervical dysplasia (i.e., CIN1) relative to women without CIN. In particular, we found that young women reported high scores across several domains, including “Self‐image” and “Partner issues and transmission.” It is likely that the majority of women with CIN had undergone colposcopic biopsy. Their experience of colposcopic biopsy is likely to have influenced their psychosocial burden, and contribute to the higher HIP scores compared with women without CIN.

Intriguingly, the mean total HIP score among women without CIN in our study (24.5) was higher than that reported in the US study (14.4) [11] lower than that reported in the South Korean study (44.98) [10], and similar to the mean scores in the Australian study (25.8) [12], the UK study (22.3) [9], and the Taiwanese study (28.2) [13] We suspect that the differences in scores might be related to the negative impact of cervical screening and gynecological examinations among women versus the impact of other HPV‐related diseases such as genital warts, differences in the healthcare systems, and cultural factors. Therefore, caution is required when comparing data across studies.

We used the HIP because it was used in prior studies in other countries [9, 10, 11, 12, 13] and is potentially more sensitive for detecting the psychosocial impact of HPV‐related genital diseases than generic quality of life questionnaires [12]. In our study, the K6 scores demonstrated the distress associated with CIN, although it was not sensitive enough to detect the psychological burden associated with CIN1, whereas the HIP scores revealed an increased psychosocial burden even among women with CIN1. This is because disease‐specific questionnaires can capture disease‐specific information across relevant aspects of their life.

We also found some potential divergence between the HIP and K6 scores because the differences in scores between women with CIN and those without CIN tended to be more pronounced for the HIP scores. We believe this may be explained by the scope of these scales. The K6 scale focuses on psychological stress and psychological distress [15, 16, 17, 18], whereas the HIP questionnaire provides a more comprehensive evaluation of the psychosocial burden associated with screening and HPV infection [11]. Although time constraints are not directly included in the HIP items, they may have an impact as an element of the psychosocial burden. Therefore, the HIP questionnaire, which captures broader psychosocial burden, is more likely to detect differences among women with or without CIN, or by grade of CIN, than the K6 scale, which purely measures psychological distress. In fact, compared to women without CIN, we observed a significant difference in the K6 scores among women with high‐grade lesions (CIN2/3), who are expected to have a greater psychological burden, but not in women with CIN1, whose psychological distress is expected to be milder.

The two major pillars of preventing cervical cancer and CIN are HPV vaccination and routine cervical screening. HPV vaccination is a critical component of this strategy for the prevention of cervical cancer and CIN and for reducing the psychological and cost burdens associated with CIN and the screening for these lesions in women. The 9‐valent HPV vaccine is particularly effective in reducing CIN in Japan because, in addition to types 16 and 18, it can prevent infection with other genotypes that cause most cases of CIN in Japanese women, such as HPV52 and HPV58 [21]. Although HPV vaccines are available and government recommendation for HPV vaccination has resumed in Japan, the vaccination coverage remains low [22, 23], contributing to a relatively high prevalence of HPV‐related diseases such as CIN. Broader vaccination coverage has the potential to reduce the overall medical burden of HPV‐related diseases, including CIN [24, 25, 26], and could therefore have a positive impact on alleviating the psychosocial burden associated with screening and diagnostic testing.

There are some limitations of this study to consider that might introduce some bias and warrant due care when interpreting the results. The participants used an online health service, and they might therefore have greater health literacy or be more willing to participate in healthcare research than the general population. The financial incentive was designed to encourage more people to participate, but many of the respondents were ultimately found to be ineligible for the main questionnaire. A large proportion of women had no record of a CIN visit within the previous 6 months; extending the look‐back period might have allowed us to identify more women with CIN. The group of women without CIN might also include some women with CIN if the relevant diagnostic/test claims were recorded prior to the look‐back period. Finally, the severity of CIN was unknown for approximately 50% of the women in this study because a relevant ICD‐10 code was not recorded in their administrative claims data. However, the HIP scores for this subgroup were numerically similar to those of women with CIN1 or CIN2/3, suggesting that this group included a mixture of women with varying severities of CIN.

In conclusion, this study of women in Japan, the first of its type, demonstrated a high psychosocial burden of CIN that was evident regardless of the severity of CIN. The psychosocial burden was already apparent in women with CIN1 and in younger women. HPV vaccination, which has evidence of reducing the number of women who develop CIN, would be expected to alleviate the burden.

## Author Contributions

**Kayo Sato:** investigation, methodology, visualization, writing – review and editing. **Nobuyuki Oshima:** visualization, writing – review and editing. **Mamiko Onuki:** visualization, writing – review and editing. **Koji Matsumoto:** visualization, writing – review and editing. **Kotoba Okuyama:** project administration, writing – review and editing, visualization.

## Funding

This work was supported by MSD K.K.

## Ethics Statement

This observational, cross‐sectional study adhered to the Japanese Ethical Guidelines for Medical and Biological Research Involving Human Subjects and the Act on the Protection of Personal Information. The study was approved by the Toukeikai Kitamachi Clinic Ethics Review Board (approval number: MSD08190; date: July 21, 2021).

## Consent

Potential participants were directed to a screening questionnaire in the Kencom application, which provided an explanation of the study, and they were required to review the explanation and check a box on the informed consent page confirming their consent to participate before they could proceed to the main questionnaire.

## Conflicts of Interest

Kayo Sato and Nobuyuki Oshima are employees of MSD K.K. Kotoba Okuyama was an employee of MSD K.K. during the conduct of the study. Mamiko Onuki received advisory fees from MSD K.K. in relation to this study. Koji Matsumoto received advisory fees from MSD K.K. in relation to this study, and honoraria for lectures from MSD K.K., Hologic Inc., and Abbott.

## Supporting information


**Table S1:** International Classification of Diseases, 10th revision, diagnostic codes.

## Data Availability

The datasets generated and analyzed during the current study are not publicly available because the participants did not give consent for sharing data with external researchers and because of contract limitations regarding the distribution of administrative data.
