# Supplementary material for: Psychosocial Burden of Women With Cervical Intraepithelial Neoplasia in Japan: Results of A Web‐Based Survey
Source: J Obstet Gynaecol Res. 2026 Mar 30;52(4):e70258. doi: 10.1111/jog.70258 (PMC13035917; doi:10.1111/jog.70258)
Supplement: Supplementary file 1 — Table S1: International Classification of Diseases, 10th revision, diagnostic codes. [file JOG-52-0-s001.pdf]

SUPPORTING INFORMATION

Psychosocial burden of women with cervical intraepithelial neoplasia in Japan: Results of a web-based survey

Kayo Sato | Nobuyuki Oshima | Mamiko Onuki | Koji Matsumoto | Kotoba Okuyama

Corresponding author: Kayo Sato. E-mail: kayo.sato@msd.com

TABLE S1 International Classification of Diseases (10th revision) diagnostic codes

**Women with CIN**
Presence of any of the following codes recorded up to 6 months before the survey
• N87.0: Mild cervical dysplasia
• N87.1: Moderate cervical dysplasia
• N87.2: Severe cervical dysplasia, not elsewhere classified
• N87.9: Dysplasia of cervix uteri, unspecified
• D06: Carcinoma in situ of cervix uteri
Providing there were no records of diagnosis or treatment for C53 Cervical cancer within 6 months after a record for any of the above codes
**Women without CIN or other diseases potentially associated with HPV infection**
No record of any of the following codes within 6 months before the survey
• N87.0: Mild cervical dysplasia
• N87.1: Moderate cervical dysplasia
• N87.2: Severe cervical dysplasia, not elsewhere classified
• N87.9: Dysplasia of cervix uteri, unspecified
• R87: Abnormal findings in specimens from female genital organs
• D06: Carcinoma in situ of cervix uteri
• K867: Surgical resection of uterine cervix
• C0-14: Head and neck cancers
• C21: Anal cancer
• C51: Vulvar cancer
• C52: Vaginal cancer
• C53: Cervical cancer
• A630: Genital warts

Abbreviations: CIN, cervical intraepithelial neoplasia; HPV, human papillomavirus.
